# Capturing experienced invisibility among people with multiple stigmatized identities: The experienced invisibility scale protocol

**DOI:** 10.1371/journal.pone.0361028

**Published:** 2026-10-30

**Authors:** Amanda K. Sesko, Alexandra N. Fisher, Monica Biernat

**Affiliations:** 1 Department of Social Sciences, University of Washington Tacoma, Tacoma, Washington, United States of America; 2 School of Philosophy, Psychology and Language Sciences, University of Edinburgh, Edinburgh, Scotland, United Kingdom; 3 Department of Psychology, University of Kansas, Lawrence, Kansas, United States of America; University of Salerno, ITALY

## Abstract

Invisibility is the experience of not being attended to, remembered, differentiated from others, or given credit for contributions. In the current registered report, we aim to validate the Experienced Invisibility Scale (EIS), a measure designed to capture experiences of invisibility, which are most likely to be experienced by people with multiple stigmatized identities. As proof of concept, we first present a pilot study testing the factor structure of the EIS, in which we found that: 1) a bifactor model (“unheard” and “unnoticed”) best represents the structure of experienced invisibility, but that calculating a total score is likely to be sufficient for most purposes, 2) the EIS is most likely to be experienced by those with multiple stigmatized identities, and 3) initial tests of convergent, discriminant, and predictive validity were supported. We then propose three studies to further validate the EIS and its links to social identities. Study 1 will distinguish the EIS from related constructs of social exclusion, objectification, rejection sensitivity, positive and negative affect, and other experiences of discrimination, test the temporal stability of the EIS, and examine relationships with well-being. Study 2 will examine whether the EIS predicts additional outcomes including health, low felt impact, perceived powerlessness, and denial of services or power. Study 3 will test hypotheses that Black women (compared to White women and Black and White men) will report experiencing more invisibility, and will show stronger relationships between invisibility and negative outcomes. We hope this work will aid scholars in better understanding invisibility as a form of discrimination.

## Introduction

*There are moments where I feel like nothing I do or will ever do will make any sort of impact on the world. Sometimes I feel very small in the whole grand scheme of things and nothing I say or do will make a difference. Sometimes it is like I can fade into the background and go about life without making a splash no matter how hard I try. Sometimes it feels like no one will listen because of my racial identity, gender, and sexual orientation.* (Black lesbian middle-class woman; authors’ unpublished data.)

Invisibility, as a form of discrimination, is the experience of not being attended to, remembered, differentiated from others, or given credit for contributions [1–4]. The focus of the current research is on *intersectional invisibility*, a unique form of discrimination, experienced by individuals with multiple stigmatized identities (e.g., Black women) [2]. As demonstrated in the quote above from a participant in our research, invisibility leaves people feeling as if they are without “impact.” In the current research, we aim to validate the Experienced Invisibility Scale (EIS) [4], a measure designed to capture experiences of invisibility so that scholars can work to better understand and reduce this relatively under-recognized, but systemic and chronic form of discrimination.

Invisibility has been discussed across disciplines, groups, and contexts, perhaps most notably by Black feminist scholars who have highlighted the role of intersections of social identities (e.g., race, gender, social class) in the socio-political erasure of Black women (e.g., in suffragist and civil rights movements) [5–9]. This erasure is captured in Sojourner Truth’s famous quote, “Ain’t I a woman?” at a woman’s rights convention in 1851, and by bell hooks [8]: “No other group in America has so had their identity socialized out of existence as have black women. We are rarely recognized as a group separate and distinct from black men, or as a present part of the larger group “women” in this culture” (p7). King [9] noted that “conceptual invisibility has led to the actual strategic neglect and physical exclusion or nonparticipation of black women” (p58).

Research on intersectional invisibility is rooted in intersectionality theory which proposes the multitude of social categories that we belong to matter; focusing on one category alone (e.g., gender) misses the experiences that accrue and are modified based on other intersecting social category memberships (e.g., race, social class, sexual identity) [5,9–11]. Intersectional theory also highlights the role of power in the experiences that occur at the intersections of social categories. Belonging to multiple groups that are lower in status affords different experiences, including discriminatory experiences, than belonging to fewer low status groups [4,12]. For example, being ignored by a colleague at work is likely experienced differently, with different outcomes, for a Black woman compared to a White woman [13–15].

Psychologists have also described invisibility experienced by other minoritized groups. For example, the invisibility of American Indians and Alaska Natives has been discussed as the absence of representation (e.g., in research, policies, and media) and through misrepresentation where groups are presented in erroneous or outdated ways (e.g., sports mascots, cartoon characters such as Pocahontas) [16, 17]. In interviews with faculty of color, Settles [15] found themes of social and professional exclusion and lack of recognition for academic achievements, and Remedios and Snyder [18] found that participants with two or more (vs. zero or one) stigmatized identities reported feeling more invisible “because of my identities (race/ethnicity, gender, age, sexual orientation, social class)” (we refer to this measure as “identity-based invisibility”).

### Being perceived by others as invisible

Psychological research on *intersectional invisibility* has largely focused on perceiver perspectives rather than the *experiences* of invisibility. For example, Sesko and Biernat [3] defined invisibility as a perceptual error, the lack of individuation or differentiation among target group members with multiple stigmatized identities. Documenting the invisibility effect, they found that White perceivers made more face recognition and statement attribution errors (confusing “who-said-what” in a group conversation) for Black women relative to White women and Black and White men.

Psychological theorizing on the causes of invisibility has also focused on the perceiver. For example, Neel and Lasseter [19] argue that *interpersonal invisibility*, treating an individual with indifference or inattention, occurs when others are regarded as irrelevant to our goals. In this model, any person, regardless of their identities, may be invisible to others if they are perceived as goal-irrelevant. Petsko and Bodenhausen’s [20] lens model does not speak directly to invisibility, but suggests that targets are stereotyped based on single identities (or an intersection of identities) that are most context-relevant. Hall et al. [21] describe how perceivers integrate stereotypes of multiple intersecting and associated categories to affect impression formation and (in)visibility.

The *intersectional invisibility* framework focuses on the unique experiences that result from the intersections of devalued identities [2]. (Low) prototypicality, or the extent to which an individual group member matches (or does not match) expected characteristics of the group [22], has been the primary theorized antecedent of intersectional invisibility [2,3,23]. For example, stereotypes of “women” tend to align more with White women (one stigmatized identity) compared to women of color (Asian, Black, Latino, Middle Eastern; two stigmatized identities), and stereotypes of racial/ethnic groups tend to overlap more with stereotypes of *men* in those groups (one stigmatized identity) [24]. Gay Black men (two stigmatized identities) are judged as less similar to the group “men” than are gay White men (one stigmatized identity) [25]. Research participants are slower to categorize the race and gender of Black women targets [26], less accurate when categorizing Black women by gender (confusing them with men) [27], and more likely to bring to mind a man than a woman when asked to think about a Black person [28]. There is evidence this pattern develops in early childhood; children as young as 5 were slower and less accurate when categorizing and Black women by gender and were less likely to attribute feminine stereotypes to Black women (compared to White and Asian women) [29].

Perceived distance between intersectional groups and their broader constituent categories also predicts the invisibility effect. In one study, participants who perceived less overlap between the stereotypes of “women” and “Black women” were more prone to face recognition errors of Black women relative to White women and Black and White men [23,30]. However, intersectionality is complex. For example, stereotypes of Asians tend to overlap more with stereotypes of Asian women than Asian men [31], and on cognitive tasks, Asian men (the less prototypical group member) tend to be the targets of recognition errors [28].

### Measuring experienced invisibility: The Experienced Invisibility Scale (EIS)

Methodologically, there has been a divide between research on *experiencing* invisibility, which has been largely qualitative in nature [e.g., 15] and the *perception* of invisibility, studied quantitatively [3]. In a recent paper, Sesko and Biernat [4] investigated whether the number of stigmatized groups to which people belong predicts (quantitatively) the experience of invisibility. They created a 13-item Experienced Invisibility Scale (EIS) based on previous quantitative and qualitative work on invisibility [3,15,23] tapping into experiences of going “unnoticed” and “unheard”, including items such as “Sometimes people who should know me don’t recognize me” and “My contributions to group discussions often go unnoticed” (rated on 1 – *not at all true*, to 7- *very true* scales; See Table 3 for all items). Across three studies they found that participants who reported more stigmatized identities (measured across various combinations of race, gender, sexual orientation, social class, non-nativity as an English speaker, first generation student status, and immigration status) were more likely to report experiencing invisibility. These experiences also predicted lower well-being, and experienced invisibility mediated the effect of number of stigmatized identities on reduced well-being. However, Sesko and Biernat [4] did not conduct a full validation of the EIS as a measure of experienced invisibility. We take on the validation goal in the current paper, in which we investigate the psychometric properties of the EIS.

In initial psychometric tests, Sesko and Biernat [4] found good internal consistency for their single-factor, 13-item EIS scale (αs from .89 − .93). The EIS was correlated with but distinct from other discrimination experiences (e.g., fear of discrimination, discrimination due to social identities) and the similar constructs of hypervisibility (tokenism, heightened scrutiny) and strategic invisibility (intentionally diverting visibility to avoid scrutiny) [15,32]. Other research has shown that invisibility can co-occur with hypervisibility and strategic invisibility [15], but while the EIS was positively correlated with these experiences, some distinctions emerged [4]: The EIS scale, but not a measure of hypervisibility, mediated the relationship between number of stigmatized identities and well-being, and strategic invisibility was a simultaneous, independent mediator of the effect of number of stigmatized identities on well-being. We further explore hypervisibility and strategic invisibility and their relationship to experienced invisibility in the current research.

### Experienced invisibility and related constructs

Everyone may experience an occasional interpersonal snub, but invisibility is likely experienced chronically by those with multiple stigmatized identities [4]. This chronicity may contribute to lower well-being [4], to feelings of low impact or powerlessness (as demonstrated in the opening-quote), fewer afforded positive life-outcomes (e.g., power, granted services, advancement and recognition at work) [2,15], and lower sense of belonging and possible selves [33].

On its face, invisibility appears to be a form of social exclusion [15,34]. Social exclusion–physical and emotional separation from others–has been conceptualized in two ways: rejection-based exclusion (where one is the recipient of negative attention) and ostracism-based exclusion (where one is ignored in some way). The latter most resembles intersectional invisibility [35]. Qualitative research has found that people who have been ostracized report feeling “invisible” [36,37], and social exclusion is reported more often by stigmatized groups [38]. Measures of ostracism often include items referencing invisibility (e.g., “In general others treat me as if I am invisible”, “In general others look through me as if I do not exist”; [39]). “Oblivious ostracism” may also occur; perceivers report not including or acknowledging someone because they literally didn’t notice they were present [40]. Further research is needed to fully disentangle invisibility and social exclusion or ostracism. In the current research we measure both constructs, expecting a positive relationship between the measures.

Invisibility is also linked to objectification [35,41], the reduction of a person to an object or thing [42,43]. Cognitive objectification is “a set of cognitive processes (early visual processing, attention, and memory) through which a person is no longer perceived as a global entity, thereby reduced to its parts akin to objects” [44], p84. For example, sexual objectification of women was evident in findings that female bodies are recognized better when presented in the context of body parts rather than whole bodies, but male bodies are recognized better when presented in the context of whole bodies (vs. body parts) [45]. Like invisibility, objectification draws attention away from internal, personal characteristics. Scholars have also likened sexual objectification to ostracism stating that “sexually objectifying a woman by ignoring and devaluing some of her personal attributes or features is akin to partial ostracism” [41], p332. In one study, women who were sexually objectified reported more feelings of ostracism, which led to lower reports of fundamental need satisfaction (measured with items including “I felt invisible,” as a negative indicator of “meaningful existence”; [41]. We also measure objectification and expect a positive, but not perfect, correlation with experienced invisibility, especially among women (the group most likely to experience objectification).

Feelings of invisibility have also been referenced in research on microaggressions, or the “subtle, stunning, often automatic, and nonverbal exchanges, which are ‘put downs’” [46], p66 that are experienced by marginalized group members. For example, in one qualitative study, ethnic minorities reported that microaggressions make them feel “trapped, invisible, and unrecognized” [47], p78. Quantitative scales measuring experienced microaggressions also reference invisibility. For example, the Gendered Racial Microaggressions Scale for Black Women [48], includes a factor labeled “silenced and marginalized,” which includes questions such as “I have felt unheard” and “my comments have been ignored” (similar to invisibility) but also less related content such as “someone challenged my authority,” and “assumed I did not have much to contribute to the conversation.” Our research recognizes invisibility as part of a broader network of discriminatory experiences Black women (and other minoritized groups) may experience.

The EIS operationalizes experienced invisibility in a manner that maps onto what we know about perceivers’ heightened memory and social categorization errors when considering members of multiple stigmatized groups (e.g., Black women): Being misremembered and unheard. This distinguishes the EIS from other measures that tap into subjective experiences of invisibility explicitly due to identities [18] or as a set of devaluing experiences tied to power and representation [48]. We expect invisibility to be part of a cluster of negative experiences; however, the narrow focus of the EIS allows for a targeted assessment of invisibility experienced as person perception errors in people’s everyday lives. This allows for greater measurement precision (reducing overlap with other constructs), and for targeted consideration of the negative consequences of *experiencing* the cognitive errors that characterize invisibility.

### Overview of current research

In the proposed research, we will examine the construct validity of the Experienced Invisibility Scale (EIS), with the hope that the scale will allow for scholars to better capture experiences of invisibility, a relatively under-recognized form of discrimination. As proof of concept, we first present a pilot study, a near replication of Sesko and Biernat [4], to test the factor structure of the EIS.

We then propose three studies to further validate the EIS and its links to social identities (measured via race, gender, sexual orientation, social class, and non-nativity as an English speaker, see Sesko & Biernat [4]). In Study 1 we will distinguish the EIS from related constructs of social exclusion, objectification, rejection sensitivity, positive and negative affect, and other experiences with discrimination; we will also assess each construct’s relationships with well-being. Study 2 will examine whether the EIS predicts additional outcomes including health, low felt impact, perceived powerlessness, and discrimination experiences related to denial of services or power.

Study 3 will replicate Study 2 but will narrow the focus to the experiences of Black women in comparison to White women, Black men, and White men. Much of the intersectional invisibility scholarship focuses on Black women [3,8,9,23], so an explicit use of our measure targeting this group at the intersection of race and gender seems appropriate. Sesko and Biernat [4] sought to measure a broad array of identities, using an additive approach to intersectionality and testing if more stigmatized identities increase experiences of invisibility (also see Remedios & Snyder [18], and Else-Quest & Hyde [49] for similar approaches). However, this approach can obscure the complexity of experiences that occur at the intersections of identity. Here we hope to begin to disentangle experiences of invisibility and its consequences by constraining our focus to Black and White women and men in Study 3. Specifically, in Study 3 we test if 1) Black women report experiencing more invisibility, and 2) if experiences of invisibility translate to worse outcomes (as measured in Study 2) for Black women, relative to White women, Black men, and White men. We will also continue to test the validity of the EIS (convergent, divergent, and predictive validity) with this focused population.

In addition, as a subsidiary test, we explore measures of hypervisibility and strategic invisibility and their relationship to experienced invisibility.

Table 1 presents an overview of primary study aims and planned statistical analyses. Data collection for Study 1 will commence shortly after registered report acceptance. Study 2 and 3 will be conducted after analyses for Study 1 are completed; minor revisions to proposed Studies 2 and 3 may be made in reference to findings from Study 1.

**Table 1:** Primary study aims and statistical analysis by study.

	Primary study aims	Statistical analyses
Pilot Study	Test factor structure of the EIS; further reduce items if necessary	CFA of EIS items Pearson-moment correlations, linear regression
	Test relationship between stigmatized identities and the EIS.* Test whether EIS measures the same construct across gender and race	Structural equation modeling Measurement invariance analyses and multigroup CFA
Study 1	Confirm the factor structure of the EIS	CFA to compare bifactor to one-factor model
	Test-retest reliability of the EIS	Pearson-moment correlation and intraclass correlation coefficient (ICC)
	Test convergent validity of the EIS	Moderate to strong Pearson-moment correlations with conceptually related established scales (i.e., social exclusion)
	Test discriminant validity of the EIS	Low to moderate Pearson-moment correlations with less conceptually related scales (e.g., rejection sensitivity)
Study 2	Test predictive validity of the EIS	Linear regression with EIS as a predictor of key outcomes (e.g., health and well-being, powerlessness)
	Test incremental validity of the EIS	Multiple linear regression predicting effects of EIS above and beyond related constructs (e.g., social exclusion)
Study 3	Test validity of the EIS across the intersections of race (Black/White) and gender (women/men).	Two-way ANOVA comparing EIS by race and gender; multiple regression with EIS, race, gender, and their interaction predicting key outcomes

Notes. *This aim will also be addressed in Studies 1 and 2, including the relationship between stigmatized identities and other measures.

### General predictions

#### Factor structure and temporal stability.

Given preliminary testing by Sesko and Biernat [4], we expect the EIS factor structure to be largely unidimensional. However, the EIS was constructed to capture experiences of being “unnoticed” (e.g., face memory errors) and “unheard” (e.g., statement misattribution) found in the literature on perceptual effects of invisibility [3,23]. To that end, a bifactor structure is likely; yet, a one-factor solution is predicted to be sufficient, as these experiences theoretically should co-occur and equally encompass broad experiences of invisibility found across variable contexts. Nonetheless, this will be explored in the Pilot Study and tested again in Study 1.

Additionally, Study 1 will examine the test-retest reliability of the EIS. We predict the EIS to demonstrate good temporal stability when administered two weeks apart. But we suspect the EIS may reflect both trait (chronic) and state (fluctuating) feelings of invisibility, and the state component may lower test-retest reliability to some extent which [50]. We will use both Pearson’s *r* to indicate the extent to which participants’ scores are rank ordered in the same way across time, and an intraclass correlation coefficient (ICC) to capture absolute agreement across measurements.

Additional primary and exploratory hypotheses are described below; primary hypotheses also are summarized in Table 2.

**Table 2:** Primary hypotheses by study.

	Pilot Study	Study 1	Study 2	Study3
H1a: Number of stigmatized identities will predict more experiences of invisibility.	✕	✕	✕	
H1b: Participants with 2 or more stigmatized identities will report higher levels on the EIS than those with 0–1 stigmatized identities.		✕	✕	
H1c: Racial/ethnic minority women will report higher levels of experienced invisibility than White women and men, and racial/ethnic minority men.	✕			✕
H2: The EIS will show strong positive relationships with social exclusion and moderate-strong positive relationships with other measures of invisibility.	✕	✕	✕	✕
H3: The EIS will show weak to moderate relationships with objectification, rejection sensitivity, positive and negative affect, experiences with other types of discrimination, and fear of discrimination.	✕	✕	✕	✕
H4a: The EIS will predict lower well-being and other health related outcomes, controlling for related variables.	✕	✕	✕	✕
H4b: The EIS will predict feelings of low felt impact, perceived powerlessness, and reported discriminatory experiences associated with being denied degrees of power or services		✕	✕	✕
H5: The association between the EIS and negative outcomes will be stronger for Black women compared to White women, Black men, and White men.				✕

#### Hypothesis 1: Relationship between stigmatized identities and the EIS.

We expect number of stigmatized identities to predict more experiences of invisibility [4,18]. We will test this both using a count variable (number of stigmatized identities to which one belongs), expecting a positive correlation with scores on the EIS (H1a), and as a categorical variable, where those with 2 or more stigmatized identities (“intersectional participants”) should report higher levels on the EIS than those with 0–1 stigmatized identities (“non-intersectional participants”; H1b; Note: every person has intersecting identities; we use the short-hand of “intersectional” and “non-intersectional” to refer to intersectionality of *stigmatized* identities, specifically). We also test this prediction across intersections of race and gender; racial/ethnic minority women should report higher levels of experienced invisibility than White women and men, and racial/ethnic minority men (H2c) [3,4].

#### Hypotheses 2–5: Convergent, discriminant, and predictive validity.

We expect the EIS to be related to, yet distinct from, other forms of discrimination and exclusionary experiences. To quantify relationship strength, we will use Pearson’s *r*s in the ranges of .10−.29, .30−.50, and >.50 to represent weak, moderate, and strong associations, respectively [51]. Demonstrating *convergent validity* (H2), we predict strong positive relationships between the EIS and social exclusion, given the connection between these constructs in the literature [35]. In addition, the EIS should be moderately-strongly related to an identity-based measure of invisibility, strategic invisibility, and hypervisibility (see Sesko & Biernat [4]). However, the EIS should show only weak to moderate associations with objectification, rejection sensitivity, positive and negative affect, experiences with other types of discrimination, and fear of discrimination (H3). In other words, while we expect experiences of invisibility to be related to these latter constructs, the EIS should be distinct from other forms of discrimination and from general rejection sensitivity or negative affect.

As in Sesko and Biernat [4], the EIS should predict lower well-being and other health related outcomes (H4a). As an extension of this prediction, in Study 1, we predict that the EIS administered at Time 1 will be associated with reduced well-being at Time 2 (two weeks later). Additionally, the EIS should predict feelings of low felt impact, perceived powerlessness, and reported discriminatory experiences associated with being denied degrees of power or services (H4b) [2]. To evidence incremental validity, we will explore whether EIS predicts these outcomes over and above similar measures (e.g., social exclusion, objectification, fear of discrimination).

Finally, in Study 3, we investigate if experiencing invisibility has the same consequences for Black women compared White women/men and Black men. While anyone may experience invisibility from time to time, low afforded power and social status are likely to magnify the impacts of being ignored and treated with indifference [12]. In this research, we argue invisibility experiences are persistent and systemic for the multiply stigmatized (in this case, Black women), engendering higher stakes [4,52]. Thus, we predict that experiencing invisibility will result in worse outcomes (lower well-being and health, powerlessness, felt impact, and greater discriminatory experiences associated with being denied services and policies) for Black women compared to White women, Black men, and White men (H5).

## Pilot study: Factor structure of the EIS

### Methods

#### Participants.

Participants were 431 psychology undergraduates at the University of Kansas, compensated with course credit. No *a priori* power analysis was used; instead, we recruited as many students as possible during the spring 2024 semester. Forty-one participants were excluded for failing either of the two attention checks leaving a sample of *N* = 390.

Treatment of participants was in line with the ethical standards of the APA. Institutional review board (IRB) approval was obtained before collection of data (University of Kansas, IRB approval # STUDY00149366).

Data files and code for all studies can be found at: https://researchbox.org/4482

With regard to gender identity, the sample included 231 women, 139 men, 5 nonbinary, 4 gender fluid, 1 who preferred to self-describe, and 10 respondents who did not disclose gender; *M* age = 19.47, *SD* = 3.23 (range 18–53). Race/ethnicity was reported as 68.97% White/Caucasian, 7.23% Black/African American, 6.15% Asian, 4.62% Hispanic/Latino/Latina, 1.28% Middle Eastern/North African, 0.77% another race/ethnicity, 0.29% American Indian or Alaska Native, 0.29% Filipino, and 11.28% who did not disclose. Seventy-seven percent identified as heterosexual, 10.51% as bisexual, 4.62% preferred to self-describe, 2.56% as homosexual, 2.31% as asexual, 2.05% as pansexual, and 3.59% did not disclose. On a 10-point scale, the average social class of the sample was 6.53 (*SD* = 1.40), whereas, when measured on a 3-point scale, 75.64% reported being middle class, 13.85% upper class, and 7.95% lower class. Twenty-two percent of the sample were international students, 8.46% spoke English as a second language, and 15.38% identified as first-generation college students.

#### Procedures.

Methods were nearly identical to those used in Sesko and Biernat [4], however we included additional measures of self-esteem and physical health. Procedures were completed online using Qualtrics.

*Experienced invisibility* was measured using the EIS ([4]; see Table 3 for items). The EIS was reduced to 12 items during initial exploratory work using data from Sesko and Biernat [4] (see Supplemental Materials for details in S1 File). S*trategic invisibility* and *hypervisibility* were measured via separate 5- and 6-item scales developed by Sesko and Biernat [4] using Settles et al.’s [15] description of the constructs, rated on 1 = *not at all true*, to 7 = *very true* scales. Strategic invisibility items included: “I often avoid situations where others may reject me”, “I often keep my interactions brief with others”, “I often distance myself from others”, and “I sometimes hide my accomplishments from others.” Hypervisibility items included: “I often feel as though everyone is watching me”, “ I often feel as though people notice my failures but ignore my accomplishments”, “I am sometimes used by others to represent the demographic group(s) to which I belong”, “There are times when I feel that things I do are scrutinized too closely by others”, “I often get noticed because I’m different from others”, and “Because there are so few people like me in many situations, I sometimes feel like a token.” We also included a single-item *identity-based invisibility* measure from Remedios and Snyder [18]: “I feel invisible because of my identities (race/ethnicity, gender, age, sexual orientation, social class),” rated on a 1 = *strongly disagree* to 7 = *strongly agree* scale. Unlike the EIS, this measure explicitly references identities allowing us to differentiate the EIS’s broad applicability in capturing experiences of invisibility described in the literature, rather than perceived *reasons* for those experiences. Sesko and Biernat [4] found initial evidence for this distinction: While the EIS and identity-based invisibility were moderately – highly correlated, only the EIS mediated the relationship between number of stigmatized identities and well-being.

**Table 3:** The Experienced Invisibility Scale (EIS).

Item No.	Item
1	My contributions to group discussions often go unnoticed.
2	Things I have said are sometimes misremembered as being said by someone else.
3	Sometimes people misremember me saying something, when it was actually said by someone else.
4	Sometimes people who should know me don’t remember details about me.
5	Sometimes people who should know me remember details about me that are untrue.
6	Sometimes people who should know me don’t recognize me.
7	People often confuse me with others.
8	I feel invisible.
*9*	*I feel that people take what I have to say seriously. (Reverse scored)*
10	I feel respected in most everyday interactions with others. (Reverse scored)
11	In conversations with others, I often feel ignored.
12	I often feel as though people don’t listen to what I have to say.
13	Sometimes people forget that they’ve met me before.

Notes. Item 9 is not included in the final scale. Instructions prompt: “We are interested in your experiences in everyday life, as you interact with others in school, at work, and in public places. Please rate how true the following statements are of your experiences in everyday life using the scale provided (1-*not at all true*, to 7-*very true*).”

To measure *well-being,* we used Su et al.’s [53] 10-items Brief Inventory of Thriving (e.g., “My life is going well”; rated on 1 = *strongly disagree* to 5 = *strongly agree* scales). *Self-esteem* was measured using Rosenberg’s Self-Esteem Scale [54] (10-items, e.g., “On the whole I am satisfied with myself”; rated on 1 = *strongly disagree* to 4 = *strongly agree scales*). *Physical health* was measured using two items: “Rate your physical health over the last two months” (1 = *poor* to 7 = *excellent*) and “During the last month, how would you rate your sleep quality overall?” (1 = *very bad* to 5 = *very good*; see Muzni et al. [55] for associations between sleep and physical and mental health).

*Experiences with discrimination* were also measured. We expected these to be correlated with, but distinguishable from, invisibility (as in Sesko & Biernat [4]). We measured *discrimination experiences* by asking participants to rate, on a scale from 1 = *never* to 7 = *all the time*, “how often do you feel that you, personally, have been discriminated against because of your . . .” a) “race, ethnicity or color?”, b) “gender or gender identity?”, c) “sexual orientation?,” and d) “social class?”; α = .65. *Fear of discrimination* (adapted from Williams et al. [56]) was also measured; participants rated the extent to which “you fear being treated in the following ways:” a) “you are treated with less respect than other people are,” b) “you receive poorer service than other people at restaurants or stores”, c) “people act as if they think you are not smart”, d) “people act as if they think you are dishonest”, and e) you are called names or insulted” (rated on 1 = *not fearful at all*, to 7 = *extremely fearful* scales); α = .85.

Demographics were measured at the end of the survey. *Number of stigmatized identities* was calculated as a count, with one point added (each) for identifying with a gender other than male, a race other than White/non-Hispanic, a sexual orientation other than heterosexual, a social class rating of 4 or lower (lower class) on the MacArthur Scale of Subjective Social Status [57] where participants indicate their standing in society on a 10-rung ladder, and non-nativity as an English speaker. Scores could therefore range from 0–5 (see Remedios & Snyder [18] for a similar calculation).

#### Data analysis plan.

We first performed a confirmatory factor analysis of the EIS scale with a bifactor model specification; this models both a general factor as well as two specific factors (feeling unheard and feeling unnoticed). Bifactor models are useful for determining whether a total scale score is sufficient or whether subscales capture meaningful additional variance, thereby helping to resolve ambiguity between unidimensional and multidimensional interpretations of test scores [58,59]. We then tested two plausible alternative models: (a) a one-factor model with all items loading onto one underlying factor, and (b) a two-factor model with two dimensions (modeled as either orthogonal or oblique). All analyses were conducted in R [60] using the lavaan [61] and SEM tools [62] packages and employed Maximum Likelihood (ML) estimation.

We evaluated model fit using five indices: the overall *χ*^*2*^*,* the Comparative Fit Index (CFI), Tucker-Lewis Index (TLI), Standardized Root Mean Square Residual (SRMR), and the Root Mean Square Error of Approximation (RMSEA). CFI and TLI are relative indices which examine the proportional improvement in fit by comparing a hypothesized model with a baseline model (typically a model where all observed variables are uncorrelated) whereas SRMR and RMSEA are absolute indices which assess how well a model reproduces the sample data [63]. For the overall *χ*^*2*^, a non-significant *p* value indicates good fit [64]. In practice, however, a non-significant *p* value for this test is rare [65]. For the CFI and TLI, a value greater than 0.90 is considered good fit, and a value of 0.95 or above is even better [63]. For the SRMR and RMSEA, values below 0.08 and 0.06, respectively, are considered good fit [63,64]. For the bifactor model, we also calculate the omega coefficient, the omega hierarchical coefficient, and the omega subscale coefficient, which indicate how much total and common variance is attributed to the general factor and specific factors, allowing for examination of whether a total scale score is justified [58,59]. Model comparisons were conducted using χ^2^ difference tests, comparing both relative and absolute fit indices, and information criteria (AIC, BIC). We then calculate Pearson-moment correlations between EIS and established scales and demographic indicators, with pairwise handling of missing data. We also conduct structural regression models to examine the extent to which number of stigmatized identities and demographic factors, like gender, race, sexual orientation, English Second Language (ESL), social class, predict latent EIS (bifactor), respectively.

We examined measurement invariance to see if our scale assessed the same construct across gender (women or men) and race (racial minority or not). We tested measurement invariance at three increasingly restrictive levels: (a) configural invariance (i.e., whether the pattern of factor loadings is the same across gender groups), (b) metric invariance (i.e., whether the magnitude of factor loadings is the same across gender groups), and (c) scalar invariance (i.e., whether the item intercepts are the same across groups). We examined change in model using ∆ *χ*^*2*^ significance tests as well as change in the CFI and RMSEA fit statistics [66,67]. Measurement invariance is considered compromised when ∆ *χ*^*2*^ tests are significant, CFI drops by more than 0.01, and RMSEA increases by more than 0.015.

Finally, we conducted a multigroup confirmatory factor analysis (MG-CFA) to examine differences in the latent means of Experienced Invisibility (EIS) across four intersectional identity groups: White Men, White Women, Racially Minoritized Men, and Racially Minoritized Women. EIS was modeled using the established bifactor structure, comprising a general factor representing shared variance across all items and two orthogonal specific factors capturing distinct dimensions of experienced invisibility. We assess latent mean differences in the general factor EIS. Group comparisons were made with White Men specified as the reference group. To ensure valid comparisons of latent means, metric and scalar invariance were tested and established by constraining factor loadings and item intercepts to be equal across all groups. Latent means were freely estimated for White Women, Racially Minoritized Men, and Racially Minoritized Women, while the mean for White Men was fixed to zero for identification. We then systematically rotated the relevant reference group to allow for mean comparisons between each pair of intersectional identities (e.g., between White Women and Racially Minoritized Women).

## Results

### Confirmatory factor analysis

As shown in Table 4, the one-factor and two-factor (orthogonal) models displayed poor fit to the data. The two-factor (oblique) model displayed acceptable fit. However, inspecting the residuals for this model indicated strongly correlated residuals between two pairs of items on the same factor, the first pair being, “Things I have said are sometimes misremembered as being said by someone else” (EIS2) and “Sometimes people misremember me saying something, when it was actually said by someone else” (EIS3) and the second pair being, “Sometimes people who should know me don’t recognize me” (EIS6) and “Sometimes people forget that they’ve met me before” (EIS13). Allowing these residual correlations to intercorrelate resulted in better model fit, ∆χ^2^ = 102.03, ∆*df* = 2, *p* < .001 (see Table 4 for additional fit metrics). These findings suggest the two-factor (oblique) measurement model is degraded when these significantly correlated residuals are not modeled. We therefore proceeded to use the two-factor (oblique) model with correlated errors (CE) as the baseline model from which to compare the fit of the bifactor model.

**Table 4:** Confirmatory model comparisons in Study 1.

Model	χ2 (df)	RMSEA	SRMR	CFI	TLI	AIC	BIC
**One-Factor Model**	381.454 (54)***	0.125	0.068	0.830	0.793	15188	15331
**Two-Factor Model (orthogonal)**	414.052 (54)***	0.131	0.228	0.813	0.772	15221	15364
**Two-Factor Model (oblique)**	210.382 (53)***	0.087	0.050	0.918	0.898	15019	15166
**Two-factor (oblique) CE**	108.347 (51)***	0.054	0.037	0.970	0.962	14921	15076
**Bifactor Model**	57.403 (42)	0.031	0.024	0.992	0.987	14888	15079

The bifactor model demonstrated excellent fit to the data (see Table 4) and demonstrated superior fit compared to the next best fitting model, the two-factor (oblique) model with correlated errors, ∆χ^2^ = 50.945, ∆*df* = 9, *p* < .001, except for the BIC which was slightly higher than for the two-factor model. Nonetheless, these results generally suggest the bifactor model (Fig 1) best represented the structure of experienced invisibility.

**Fig 1:**
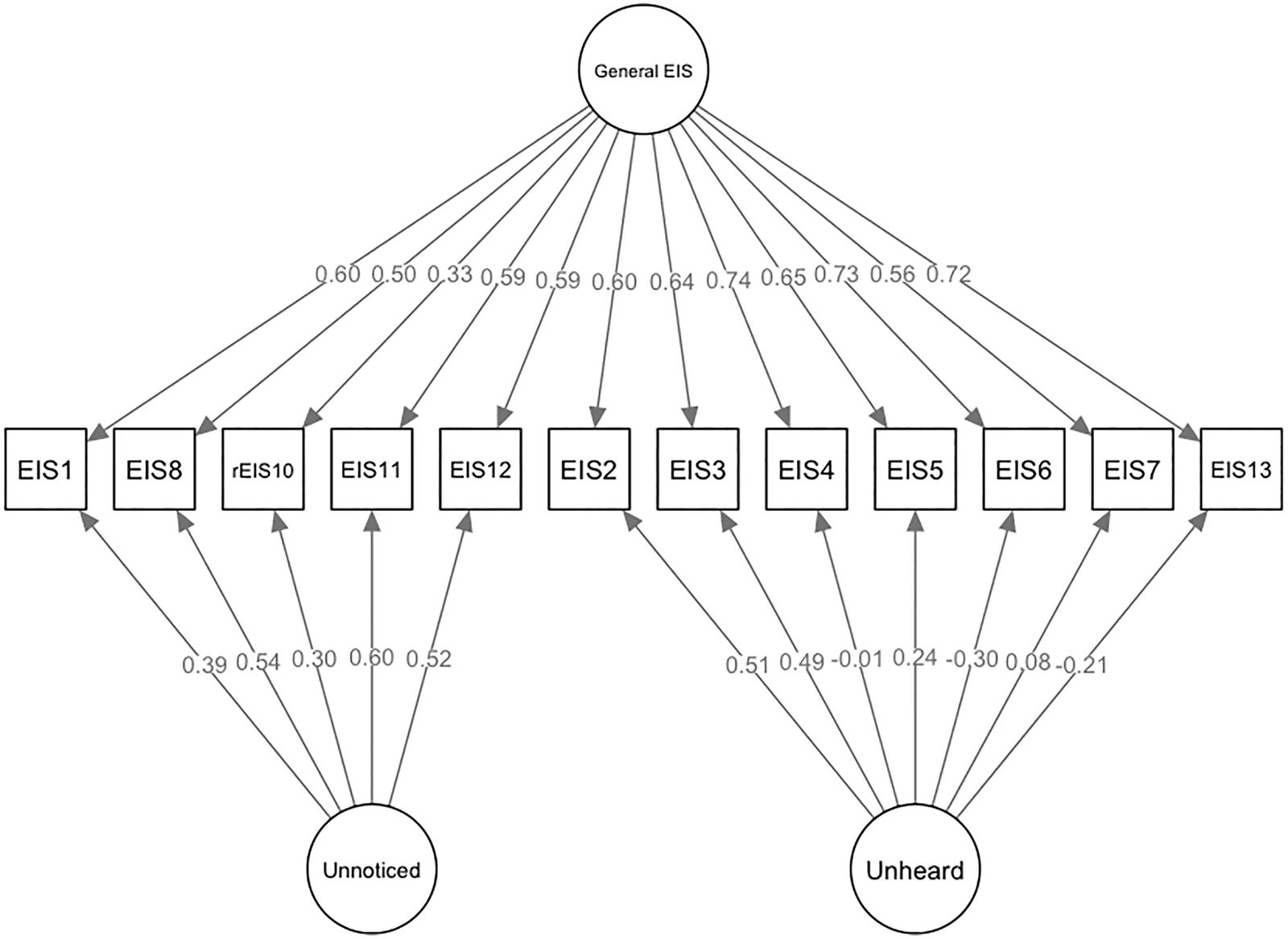
Bifactor model of experienced invisibility.

### The bifactor model of experienced invisibility

Table 5 presents factor loadings, sources of variance, and reliability estimates for the general factor and two specific factors (unnoticed and unheard). Most items had strong loadings (> .40) on the general factor except for EIS10 (.33) and generally had stronger factor loadings on the general than the specific factors (exceptions being EIS8 and EIS11). All factor loadings on the general and unnoticed factor were significant. However, the unheard factor had two nonsignificant factor loadings (EIS4 and EIS7) after accounting for the general EIS factor.

**Table 5:** Standardized factor loadings and sources of variance in experienced invisibility.

	One-factor	Two-Factor (Oblique)			Bifactor	
Indicator	General EIS	Unnoticed	Unheard	General EIS	Unnoticed	Unheard
EIS1	.71***	.72***	–	.59***	.39***	–
EIS8	.65***	.72***	–	.50***	.54***	–
EIS10	.42***	.45***	–	.33***	.30***	–
EIS11	.75***	.83***	–	.59***	.60***	–
EIS12	.73***	.79***	–	.59***	.52***	–
EIS2	.58***	–	.60***	.60***	–	.51***
EIS3	.59***	–	.63***	.64***	–	.49***
EIS4	.71***	–	.76***	.74***	–	−.01
EIS5	.63***	–	.68***	.65***	–	.24***
EIS6	.63***	–	.64***	.73***	–	−.30***
EIS7	.54***	–	.56***	.56***	–	.08
EIS13	.64***	–	.65***	.73***	–	−.21**
% total variance	.41	.20	.22	.34	.09	.05
% common variance		.47	.53	.71	.18	.11
	ω = .89	ω = .84	ω = .80	ω = .91	ω = .71	ω = .16
				ω_h_ = .82	ω_s_ = .38	ω_s_ = .02

*Note.* ω = omega coefficient; ω_h_ = omega hierarchical; ω_s_ = omega subscale. ***p = .001, **p = .01.

The general factor explained 34% of the total variance, and the specific unnoticed and unheard factors explained 9% and 5% of the total variance, respectively. Additionally, the general factor accounted for 71% of the common variance. The value of ω_h_ (omega hierarchical) indicated that 82% of the variance of the composite (general + specific factors) score in experienced invisibility was attributable to the general factor. Omega hierarchical for the subscale scores (ω_s_), which indicates the reliability of the specific factors after accounting for the general factor, was poor for both the unnoticed factor (.38) and for the unheard factor (.02). Moreover, 55% of the reliable variance in unnoticed and 18% of the reliable variance in unheard was independent of the general factor (ω_s_/ω). These results suggest that the specific factor scores for unheard and unnoticed are unreliable after controlling for the general factor score.

Nonetheless, the bifactor model accounted for 48% of the total variance whereas the two factor and one-factor model accounted for 42% and 40% of the total variance, respectively. Taken together, these results suggest that, although a bifactor model best represents the structure of experienced invisibility, calculating a total score is likely to be sufficient for most purposes [59]. In the following predictive and measurement invariance analyses, we report our results using the general EIS factor and specific factors from the bifactor model but will once again test the fit of the bifactor model compared to the one- and two-factor models in Study 1.

### Correlates of experienced invisibility

Tables 6 and 7 present descriptive information and Pearson-moment correlations among all measured variables. EIS was positively correlated with strategic invisibility, hypervisibility, identity-based invisibility, experiences of discrimination, fear of discrimination, and number of stigmatized identities. EIS was negatively correlated with well-being, self-esteem, physical health, and sleep quality. In terms of demographic variables, EIS was positively correlated with being a woman or gender minority, being a sexual minority, speaking English as a second language (ESL), and negatively correlated with socioeconomic status.

**Table 6:** Means, standard deviations, and correlations with confidence intervals for all measures.

Variable	*M*	*SD*	1	2	3	4	5	6	7	8	9	10
1. EIS	2.84	0.98										
2. Strategic Invisibility	3.99	1.13	.53**									
			[.46, .60]									
3. Hypervisibility	3.19	1.11	.51**	.40**								
			[.43, .58]	[.31, .48]								
4. Identity-based Invisibility	2.25	1.42	.44**	.33**	.45**							
			[.35, .51]	[.24, .42]	[.36, .52]							
5. Well-Being	3.86	0.63	−.44**	−.41**	−.14**	−.25**						
			[-.52, -.36]	[-.48, -.32]	[-.24, -.04]	[-.34, -.15]						
6. Self-Esteem	2.85	0.41	−.42**	−.41**	−.16**	−.29**	.75**					
			[-.50, -.33]	[-.49, -.32]	[-.26, -.06]	[-.38, -.19]	[.71, .79]					
7. Physical Health	4.81	1.35	−.33**	−.26**	−.14**	−.18**	.44**	.41**				
			[-.41, -.24]	[-.35, -.16]	[-.23, -.04]	[-.27, -.08]	[.36, .52]	[.33, .49]				
8. Sleep	2.96	1.07	−.31**	−.26**	−.22**	−.23**	.38**	.31**	.38**			
			[-.40, -.22]	[-.35, -.16]	[-.32, -.13]	[-.33, -.14]	[.29, .46]	[.22, .40]	[.30, .47]			
9.Discrimination	2.00	0.90	.36**	.19**	.38**	.50**	−.24**	−.24**	−.20**	−.27**		
			[.27, .44]	[.09, .28]	[.29, .46]	[.42, .57]	[-.33, -.14]	[-.33, -.15]	[-.29, -.10]	[-.36, -.18]		
10. Fear of Discrimination	3.20	1.48	.35**	.24**	.31**	.36**	−.26**	−.34**	−.24**	−.22**	.36**	
			[.26, .44]	[.14, .33]	[.22, .40]	[.27, .44]	[-.35, -.17]	[-.42, -.25]	[-.33, -.15]	[-.31, -.12]	[.28, .45]	
11. Number of Stigmatized IDs	1.38	1.03	.21**	.18**	.23**	.28**	−.26**	−.25**	−.27**	−.30**	.38**	.23**
			[.11, .30]	[.08, .28]	[.13, .32]	[.19, .37]	[-.35, -.16]	[-.34, -.15]	[-.36, -.17]	[-.39, -.21]	[.29, .47]	[.13, .32]

*Notes. M* and *SD* are used to represent mean and standard deviation, respectively. Values in square brackets indicate the 95% confidence interval for each correlation. The confidence interval is a plausible range of population correlations that could have caused the sample correlation [68]. *indicates *p* < .05. ** indicates *p* < .01. EIS = Experienced Invisibility Scale, IDs = Identities.

**Table 7:** Demographic means, standard deviations, and correlations with confidence intervals.

Variable	*M*	*SD*	1	2	3	4	5	6
1. EIS	2.84	0.98						
2. Nonmale	0.63	0.48	.16**					
			[.06, .26]					
3. Sexual Minority	0.23	0.42	.11*	.07				
			[.01, .21]	[-.03, .17]				
4. Race Minority	0.34	0.47	.07	−.11*	.03			
			[-.03, .16]	[-.21, -.01]	[-.07, .13]			
5. ESL	0.09	0.28	.15**	.00	.10	.35**		
			[.05, .24]	[-.10, .10]	[-.00, .20]	[.26, .44]		
6. Social Class	6.53	1.40	−.15**	−.08	−.20**	−.26**	−.16**	
			[-.25, -.05]	[-.18, .02]	[-.29, -.10]	[-.35, -.17]	[-.26, -.06]	
7. Age	19.47	3.23	.01	−.14**	.03	.01	.01	−.11*
			[-.09, .11]	[-.23, -.04]	[-.07, .13]	[-.09, .11]	[-.09, .11]	[-.21, -.01]

*Note. M* and *SD* are used to represent mean and standard deviation, respectively. Values in square brackets indicate the 95% confidence interval for each correlation. The confidence interval is a plausible range of population correlations that could have caused the sample correlation [68]. *indicates *p* < .05. ** indicates *p* < .01. ESL = English as a second langauge.

Next, we simultaneously regressed latent experienced invisibility (general and specific factors) on observed variables gender, race, sexual orientation, ESL, and social class. Only speaking English as a second language, *b* = .39, *B* = .15, *SE* = .16, *z* = 2.430, *p* = .015, emerged as a significant predictor of general score EIS. However, being a woman or gender minority, *b* = .20, *B* = .19, *SE* = .07, *z* = 2.955, *p* = .003, being a sexual minority, *b* = .18, *B* = .15, *SE* = .07, *z* = 2.373, *p* = .018, and social class, *b* = −.09, *B* = −.24, *SE* = .02, *z* = −3.517, *p* < .001, predicted latent scores on the Unnoticed specific factor. There were no significant predictors for the Unheard specific factor.

We regressed latent experienced invisibility (general and specific factors) on number of stigmatized identities. Number of stigmatized identities did not predict general factor EIS, *b* = .02, *B* = .04, *SE* = .04, *z* = 0.582, *p* = .561, nor did it predict the Unheard specific factor, *b* = .03, *B* = .18, *SE* = .03, *z* = 0.955, *p* = .340. However, number of stigmatized identities did predict higher scores on the Unnoticed specific factor, *b* = .21, *B* = .34, *SE* = .04, *z* = 4.748, *p* < .001.

### Measurement invariance across gender and race

The results supported configural, loading, and intercept measurement invariance across gender and race (see Table 8), allowing us to meaningfully compare latent means in experienced invisibility and correlations with experienced invisibility across intersectional groups. We therefore tested for latent mean differences in general and specific factors EIS across the intersection of gender and race.

**Table 8:** Results of measurement invariance testing bifactor model across gender and race in the pilot study.

Model	χ2 (df)	∆ χ2 (df)	CFI	∆CFI	RMSEA	∆RMSEA
Gender						
**Configural**	108.89 (84)		0.986		0.040	
**Loading**	134.69 (105)	25.81 (21)	0.983	−0.003	0.039	−0.001
**Intercept**	141.79 (114)	7.10 (9)	0.984	0.001	0.036	−0.003
**Race**						
**Configural**	115.93 (84)		0.984		0.044	
**Loading**	148.13 (105)	32.19 (21)	0.978	−0.006	0.046	0.002
**Intercept**	164.65 (114)	16.52 (9)	0.974	−0.004	0.048	0.002

### Multigroup analysis across the intersection of gender and race

#### General factor EIS.

Relative to White men (latent mean fixed at 0), racially minoritized women scored significantly higher on general factor EIS, *M* = 0.30, *SE* = 0.14, *z* = 2.19, *p* = .029, whereas White women did not differ significantly from White men on general factor EIS, *M* = 0.07, *SE* = 0.10, *z* = 0.72, *p* = .471, nor did racially minoritized men, *M* = −0.08, *SE* = 0.13, *z* = −0.61, *p* = .545 (see Fig 2).

**Fig 2:**
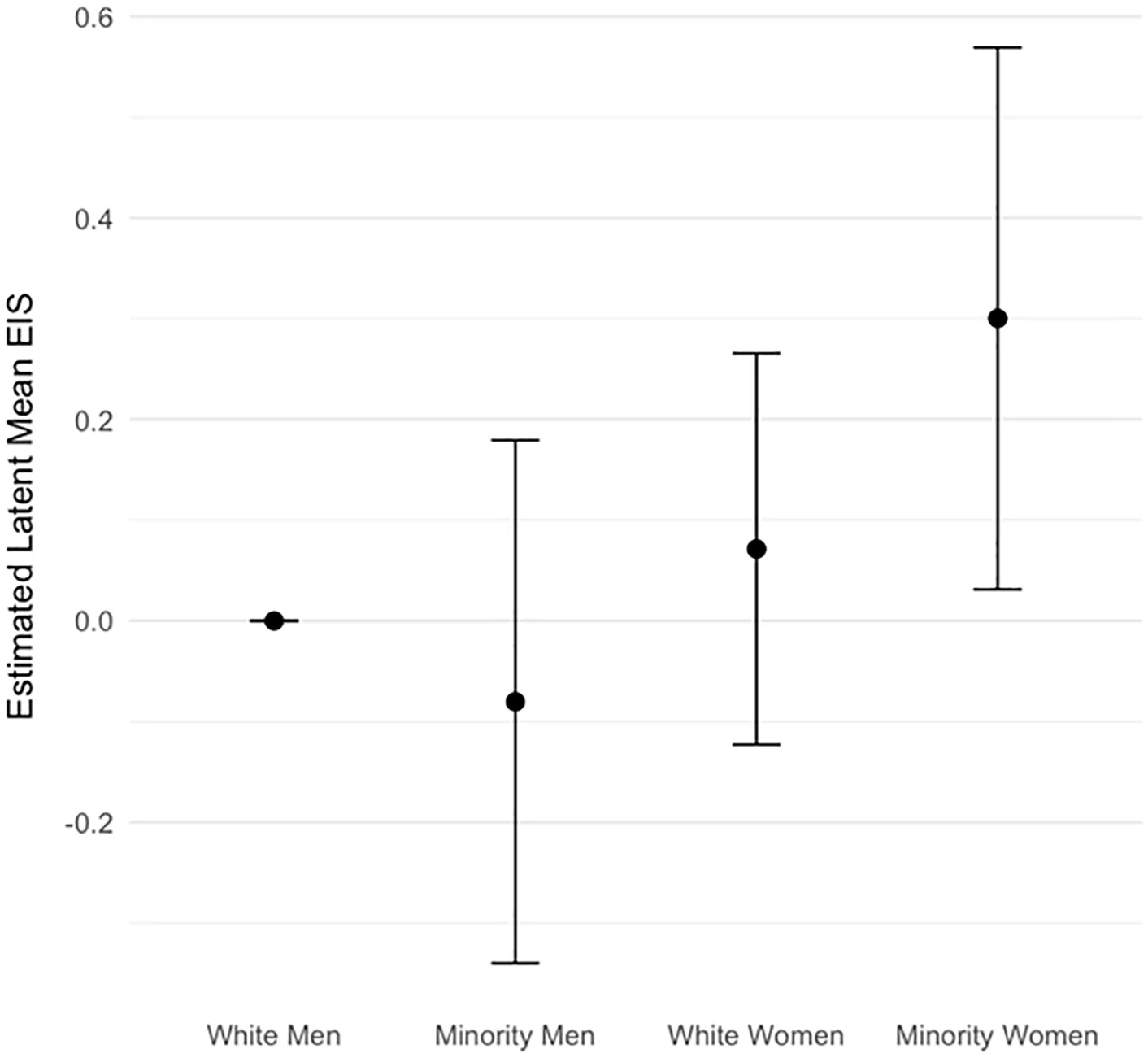
Latent mean experienced invisibility (general factor) across intersections of gender and race in the pilot study.

Racially minoritized women also reported higher general factor EIS than racially minoritized men, *M* = 0.38, *SE* = 0.16, *z* = 2.408, *p* = .016, and marginally higher general factor EIS than White women, *M* = 0.23, *SE* = 0.13, *z* = 1.782, *p* = .075. Finally, White women and racially minoritized men did not differ in general factor EIS, *M* = 0.15, *SE* = 0.12, *z* = 1.228, *p* = .219.

#### Unnoticed specific factor.

Relative to White men, racially minoritized women scored significantly higher on the Unnoticed specific factor, *M* = 0.32, *SE* = 0.12, *z* = 2.740, *p* = .006. White women did not differ from White men on the Unnoticed specific factor, *M* = 0.12, *SE* = 0.08, *z* = 1.496, *p* = .135, nor did racially minoritized men differ from White men, *M* = −0.13, *SE* = 0.09, *z* = −1.1353, *p* = .176. Racially minoritized women also had higher Unnoticed factor scores than racially minoritized men, *M* = 0.45, *SE* = 0.14, *z* = 3.335, *p* = .001, and marginally higher Unnoticed factor scores than White women, *M* = 0.20, *SE* = 0.11, *z* = 1.844, *p* = .065. White women had higher Unnoticed factor scores than racially minoritized men, *M* = 0.35, *SE* = 0.10, *z* = 2.504, *p* = .012.

#### Unheard specific factor.

Racially minoritized women did not differ from White men in Unheard factor scores, *M* = −0.25, *SE* = 0.16, *z* = −1.506, *p* = .132. White women also did not differ from White men on the Unheard specific factor, *M* = 0.08, *SE* = 0.12, *z* = 0.643, *p* = .520, nor did racially minoritized men differ from White men, *M* = 0.10, *SE* = 0.13, *z* = 0.738, *p* = .460. However, racially minoritized women had lower Unheard factor scores than racially minoritized men, *M* = −0.35, *SE* = 0.17, *z* = −1.990, *p* = .047, and lower Unheard factor scores than White women, *M* = −0.32, *SE* = 0.16, *z* = −2.009, *p* = .045. White women and racially minoritized men did not differ in Unheard factor scores, *M* = 0.02, *SE* = 0.13, *z* = 0.181, *p* = .857.

### Pilot study discussion

The Pilot Study results suggest that a bifactor model best represents the structure of experienced invisibility, but that calculating a total score is likely to be sufficient for most purposes. As expected, these data generally mirror past operational definitions of invisibility where voices and faces were poorly differentiated and misremembered (factors of “unheard” and “unnoticed”) [3]. Indeed, we expect both types of perceptual errors to be experienced, and have no theoretical or empirical evidence to suggest that one is more representative of invisibility than the other. Rather we suggest they co-occur; if someone is less likely to be noticed, they are also less likely to be heard, and vice versa. Situational constraints likely afford moments where one type of experience is more likely than the other, but the EIS is meant to capture general experiences of invisibility across a broad array of situations. In Study 1, we aim to confirm this factor structure.

When testing for group differences across specific factors, results were nuanced. As expected, the Unnoticed factor was positively correlated with number of stigmatized identities, and specifically with being a woman or gender minority, being a sexual minority, speaking English as a second language (ESL), and being lower in socioeconomic status. While these patterns were not found for the Unheard factor, a test of latent mean differences across the intersection of gender and race showed that relative to White men, racially minoritized women, reported significantly higher EIS general factor and Unnoticed factor scores, whereas White women and racially minoritized men did not. In addition, racially minorized women reported higher general factor EIS scores than racially minorized men.

These findings go beyond reported results in Sesko and Biernat [4] in several ways. First, they highlight potential nuances between factors. Second, the multigroup analysis across the intersections of race and gender is new to this research and supports the notion that racial minority women are most subjected to invisibility, relative to their White women and racial minority counterparts. Unexpectedly, ESL emerged as the single identity predictor of the general EIS score. At first glance, this could suggest the EIS’s emphasis on language and communication in the “unheard” factor (e.g., “In conversations with others, I often feel ignored”) is tapping into experiences of fluency. However, ESL did not hold as predictor of the unheard factor (nor the unnoticed factor). In addition, there is reason to believe this result may be unreliable: across the three studies presented in Sesko and Biernat [4], ESL was significantly correlated with the EIS in only one study (and instead, most consistently correlated with lower social class). We therefore hesitate to draw strong conclusions about this finding. Collectively the Pilot Study results suggest the EIS measures experiences that are most likely to be found at the intersections of stigmatized identities. Group differences across the specific factors of Unnoticed and Unheard will be further explored in Study 1.

The Pilot Study also confirmed initial tests of convergent, discriminant, and predictive validity. Showing convergent validity, the EIS demonstrated moderate-strong positive associations with other measures of invisibility including strategic invisibility, hypervisibility, and identity-based invisibility. Showing discriminant validity, the EIS was related to experiences of discrimination and fear of discrimination, but only moderately so, and less so than other measures of invisibility. As evidence of predictive validity, the EIS was negatively correlated with well-being, self-esteem, physical health, and sleep quality.

## Study 1: Is the EIS distinguishable from related constructs and stable over time?

In Study 1, we propose to confirm the factor structure of the EIS, continue to test the relationship between stigmatized identities and the EIS, and to further test convergent and discriminant validity. In addition, Study 1 extends psychometric testing in several ways. First, we test the temporal stability of the EIS administered two weeks apart via test-retest reliability analyses. The longitudinal design, also allows us to test if the EIS measured at Time 1 predicts reduced well-being at Time 2. Second, we continue to test if number of stigmatized identities as a count variable (accumulated number of stigmatized identities) is positively related to scores on the EIS (H1a) and, whether those with 2 or more stigmatized identities (“intersectional participants”) report higher levels on the EIS than those with 0–1 stigmatized identities (“non-intersectional participants”; H1b). Third, and central to this study, Study 1 tests convergent and discriminant validity with the additional constructs of social exclusion, objectification, rejection sensitivity, and positive and negative affect (H2 – H4b); Tables 1 and 2 summarize the contributions of each study.

### Methods

#### Participants.

Participants will be recruited via Prolific and paid $2.50 for completion of Time 1 measures and $1.25 for Time 2. Power analyses were conducted in R using *semPower* [69] and *pwr* [70]. For the bifactor CFA, an a-priori RMSEA power analysis with df = 42, α = .05, power = .80, and RMSEA = .05 indicated a required sample size of 269. However, for H1a (regressing EIS on number of stigmatized identities), we planned for the smallest effect of interest *r* = .15 (*f*^2^ = .023), chosen conservatively based on the pilot study’s correlation coefficient (*r* = .21). The required sample size based on this effect size, an alpha of .05, and 80% power was 350. A sample of 350 would therefore satisfy both thresholds and provide adequate power for our planned analyses. We have chosen to use crowdsource sampling (for this, and all subsequent studies) to diversify the pool beyond a university sample and to optimize sampling N. Sesko and Biernat [4] found similar effects across both university and crowdsource samples, suggesting the EIS captures experiences that are generalizable across these sampling techniques.

For all proposed studies, treatment of participants will be in line with the ethical standards of the APA; IRB approval has been obtained (University of Washington, IRB approval # STUDY00022824; MOD00026043).

For all proposed studies, two attention check items will be used; participants will be asked to answer in specific ways to prompts and wrong answers to either one (or both) will result in exclusion. In addition, participants who complete the entire survey in less than 3 minutes or who complete 50% or less of the survey or fail to answer the demographics questions will be deleted.

#### Procedures.

Study 1 will be completed across two time points. At Time 1 participants will complete an on-line survey (using Qualtrics) in which all of the constructs described below will be measured. To assess test-retest reliability, participants who complete Time 1 will be prompted two weeks later to complete Time 2 where EIS and well-being will be measured again. Time 2 measures will also include hypervisibility and strategic invisibility for a comparable temporal stability test.

*Experienced invisibility*, *strategic invisibility, and hypervisibility*. *Experienced invisibility* will be measured using the EIS [4], including the 12 items identified in our pilot study and previous work.

*Strategic invisibility hypervisibility* will be measured using the same two 5- and 6- item (respectively) scales developed by Sesko and Biernat [4] used in the Pilot Study.

*Social exclusion*. Social exclusion will be measured using the *General Belongingness Scale* (GBS) [71] which includes 12-items measuring *acceptance/inclusion* (e.g., “When I am with other people, I feel included”) and lack of *rejection/exclusion* (e.g., “I feel like an outsider”; items are reverse scored, higher numbers = lack of rejection/exclusion; rated on 1-*strongly disagree* to 7-*strongly agree* scales). Given higher numbers = higher belonging/lower exclusion, we expect a strong negative relationship with this scale and experiences of invisibility.

In addition, we will also assess the frequency of experiencing ostracism using the four-item *Ostracism Short Scale* (OSS) [72]. Participants will be asked how often they experienced the following occurrences during the last two months: “Others ignore me”, “Others exclude me from conversations”, “Others treat me as if I was not there at all”, and “Others do not invite me to activities” (rated on 1 = *never* to 7 = *always scales*).

*Objectification*. Body evaluation (11-item; e.g., “How often have you noticed someone leering at your body”) and sexual objectification (4-item; e.g., “How often have you experienced sexual harassment (on the job, in school, etc.)”) and will be measured using the Interpersonal Sexual Objectification Scale (ISOS) [73], rated on 1 = *never* to 5 = *almost always* scales.

*Rejection sensitivity*. Rejection sensitivity, defined as “undue and excessive awareness of, and sensitivity to, the behaviour and feelings of others” and individuals who score high on this trait, are thought to be “preoccupied with their interpersonal relationships, vigilant to the behaviour and moods of others, and overly sensitive to the vicissitudes of any interpersonal interaction” [74, p342]. In order to rule out that those who score high on the EIS are simply more attended to rejection, we will measure rejection sensitivity with two factors of the interpersonal sensitivity measure (IPSM) [74] most related to the construct of invisibility (relative to other factors of separation anxiety, timidity, and fragile-inner self): *Interpersonal awareness* (7-items; e.g., “I worry about the effect I have on other people”)*,* and *need for approval* (8-items; e.g., “After a fight with a friend, I feel uncomfortable until I have made peace”), rated on 1 = *very unlike* to 4 = *very like* scales.

*Positive and negative affect.* To distinguish experiences of invisibility from general experiences of positive and negative affect, participants will take the 20-item Positive and Negative Affect Schedule [75] in which participants will indicate if they generally feel a series of feelings and emotions (e.g., positive affect = interested, excited, strong, and negative affect = distressed, upset, irritable; rated on 1 = *very slightly or not at all* to 5 = *extremely* scales).

*Well-being.* To be consistent with Sesko and Biernat [4] and our Pilot Study, *well-being* will again be measured using the BIT [53].

*Experiences with discrimination*. Experiences with discrimination will be measured using the same questions as in the Pilot Study.

*Number of stigmatized identities.* Demographics will be measured at the end of the study and will be the same as in the Pilot Study. However, acknowledging that other identities than previously measured are likely to matter, we will also explore the role of disability as a stigmatized identity in experienced invisibility. Disability research has established links with feelings of social isolation and loneliness [76] but experienced invisibility as operationalized here has yet to be examined. We will initially measure number of stigmatized identities precisely as in the Pilot Study and in Sesko and Biernat [4] (possible range from 0–5), but we will conduct additional analyses that include disability identity as well (possible range from 0–6). However, because disabilities encompass a vast array of identities and experiences, some more visible than others, some lifelong and some more episodic, experiences of invisibility due to disabilities are also likely to vary [77]. To control for this possibility, participants will first be asked to if they “identify as a person with a disability” (yes/no), if “yes” then they will be asked to choose if their disability status is “hidden” or “visible” and to select an impairment type from the following list: physical, psychiatric/mental, learning, sensory, chronic health condition, or self-described [78].

#### Planned analyses.

We will follow the same procedures as the Pilot Study to test the fit of the Bifactor model of EIS compared to a one-factor model. If the Bifactor (or one-factor) model, again demonstrates high internal consistency (Cronbach’s α or ω ≥ .80), we will conduct the remaining analyses using an EIS composite (average) score in observed, rather than latent, space. This approach is more practical for the current design—reducing estimation complexity and improving power—while maintaining theoretical and empirical comparability with the latent model [79].

Temporal stability of EIS will be assessed by calculating both Pearson’s *r* and a two-way mixed effects intraclass correlation coefficient (ICC). Reporting both statistics will allow us to evaluate the relative stability (Pearson’*s r*) as well as agreement in absolute scores (ICC) over time.

To test H1a, EIS will be regressed on the number of stigmatized identities (0–5) as a continuous predictor (*p*-value < .05). To test H1b, estimated marginal means will be computed for each level (0–5), and a planned, proportionally weighted contrast will test whether participants with two or more identities have higher predicted EIS scores than those with zero or one identity. The inferential test will be the model-based mean difference between the 2+ and 0−1 groups, with a one-sided *p*-value (α = .05) and 95% confidence interval. To test H2-3, we will conduct Pearson-moment correlations (*p* < .05), with a Pearson’s *r* of .10−.29, .30−.50, and >.50, representing a weak, moderate, and strong association, respectively. H4a and H4b will be tested using linear regression with EIS predicting each of the well-being and discrimination outcomes, respectively. As part of H4a, we will also use multiple linear regression to examine whether EIS at Time 1 predicts well-being at Time 2, controlling for well-being measured at Time 1.

## Study 2: What does the EIS predict?

Study 2 will include all the same procedures and predictions as Study 1 (see Table 2), but additional constructs of health and well-being, powerlessness, felt impact, and denial of degrees of power or services will be measured to further examine the predictive validity of the EIS (specifically, H4b).

### Methods

#### Participants.

Using a different crowdsourced database than Study 1, participants will be recruited via CloudResearch and paid $2.50. We planned our sample size around H4a and H4b, regressing key well-being and discrimination-related outcomes on EIS, and testing the predictive ability of EIS over and above other related measures (e.g., social exclusion). We planned for a small-to-moderate effect of interest *r* = .20−30 (*f*^2^ = .042−.046), chosen conservatively based on the correlation coefficient between EIS and Discrimination in the Pilot (*r* = .36). The required sample size based on the smaller effect (*r* = .20), an alpha of .05, one covariate, and 80% power was 190 participants. We aim to collect data for 250 participants to account for possible exclusions.

#### Procedures.

All procedures will be the same as Study 1 with the addition of the following measures:

*Health and well-being*. Health and well-being will be measured in a variety of ways. The link between subjective well-being and the EIS has been established in Sesko and Bierant [4] and the pilot study reported here, using the Comprehensive and Brief Inventories of Thriving [53]. In Study 2 we will measure well-being using a different, but related part of the construct using the 5-item Overall Life Satisfaction Scale [80] (e.g., “I am satisfied with my life”), rated on 1 = *strongly disagree* to 7 = *strongly agre*e scales. This measure is “narrowly focused to assess global life satisfaction and not tap related constructs such as positive affect or loneliness” [80, p71]. To distinguish the use of this new measure, we refer to it as *life satisfaction.* In addition, participants will be asked the same questions as in the Pilot Study assessing *self-esteem* [54] and the two items capturing *physical health.* One additional item will be included as an objective indicator of physical health: “How often did you go to a doctor/medical provider this year?” (1-*not at all*, to 7-*very often* scale).

*Powerlessness*. To measure powerlessness, participants will rate their p*erceived powerlessness* on a 9-item scale developed by Lim et al. [81]. This scale was designed to capture experiences of adolescents in a context where educational and economic opportunities were limited; it was based on generalized powerless measures [82, 83], but includes additional items related to structural inequities (e.g., at work, in school, and in political participation) that we also expect to be relevant to invisibility in adults. This scale has three factors: present perceived powerlessness (e.g., “Sometimes I feel that I am being pushed around in life, “When I have a problem, I do not feel confident I can solve it,” and “At school or work, I do not always speak up when I have something to say because I do not think anyone will listen to me”), future perceived powerlessness (e.g., “I do not think that my education has prepared me to achieve a successful career”, “I do not think about voting in the future because politicians are not interested in helping to improve the situation”, “There is no point in trying to change things for the better because no one cares or wants to help”, and “Working hard in school or on the job does not guarantee better opportunities later on”), and financial perceived powerlessness (“I worry about money because jobs for me or my family members are not easy to find or keep” and “Saving money is hard to do in my household because we already have a hard time paying the bills”). Although Lim et al. [81] used 1–4 rating scales (strongly agree – strongly disagree), we will use 1 = *strongly disagree* to 5 = *strongly agree* scales to maintain consistency with other measures.

*Felt impact*. To capture the feeling of “felt impact” often described by participants when discussing invisibility (e.g., in the opening quote) we developed questions to assess perceived impact across life domains. Participants will be asked to indicate how true (1 = *not at all true* to 7 = *very true*; higher numbers = more impact) the following statements are: “My family would not function well without me,” “My co-workers would be at a loss if I were not around,” “My friendship group would fall apart if I were not around,” and “My peers would not succeed as well without me.”

*Denied degrees of power or services*. To assess the extent to which experiencing invisibility is associated with granted power and services [2], participants will complete a (Yes/No) checklist assessing if they have experienced in the past year a series of negative events: 1) “I have been denied or looked over for an opportunity at work or school”, 2) “I have been denied membership into an organization”, 3) “In health care settings my symptoms have been dismissed”, 4) “In health care settings my preferences are ignored” (items 3–4 adapted from Okoro et al. [84]), 5) “I have been denied a financial product or service”, 6) “I have been denied a rental application”, 7) “At a bar/restaurant, I have had to wait longer than others to get served”, 8) “At a store, workers have not been helpful to me”, and 9) “Policies written to protect the rights of individuals, have failed to protect me.” A total score will be calculated by summing the number of times participants answer “yes” (scores ranging from 0–9).

#### Planned analyses.

Planned analyses for H1ab-H4ab will be the same as Study 1 except for H4a where we will use multiple linear regression to examine the predictive ability of EIS when controlling for related constructs such as social exclusion and rejection sensitivity. To do this, we will systematically regress key well-being outcomes (i.e., health) on EIS and each related control variable (i.e., social exclusion), sequentially.

## Study 3: The EIS and race × gender intersectionality

Study 3 will replicate Study 2, but with a specific focus on the intersections of race (Black, White) and gender in experiences of invisibility for Black women compared to White women, Black men, and White men (testing H5).

### Methods

#### Participants.

As in Study 1, Participants will be recruited via Prolific, and paid $2.50. However instead of focusing on experiences of multiple stigmatized identities broadly, in Study 3 we will return to investigating invisibility among Black women relative to White women, Black men, and White women in particular. Monte Carlo power simulations (*n* = 1000) were conducted using estimates from the 3-way interaction of EIS, gender, race predicting discrimination experiences in our pilot study (*b* = 0.42, *SE* = 0.20, *p* < .05), which indicated an implied Cohen’s f^2^ of .012 (H5). Based on these simulations, a sample size of 520 (130 per group) would provide > .80% power to detect an interaction of this magnitude or greater.

#### Procedures.

Procedures for Study 3 will be identical to Study 2. The one exception will be measurement of stigmatized identities. Because Study 3 focuses on Black and White women and men, instead of a count variable we are instead interested in experiences of invisibility of Black women compared to Black men and White women and men. In addition, social class and sexual orientation will be measured as before to explore interactions with race and gender.

#### Planned analyses.

To test H1c, we will conduct a 2 (Race: Black, White) x 2 (Gender: women, men) factorial ANOVA with experienced invisibility as the dependent variable. Upon a significant interaction between race and gender (*p* < .05), we will conduct pairwise comparisons to test whether Black women score higher on EIS relative to White women, Black men, and White men (*p* < .05).

H2-H4ab will use the same planned analyses as in Study 1 and 2.

To test H5, we will use multiple linear regression to examine whether the association between EIS and negative outcomes is stronger for Black women than for the other three groups. Each outcome will be regressed on EIS (standardized), race (0 = White, 1 = Black), gender (0 = Women, 1 = Men), and their interactions. The focal test is the 3-way interaction between EIS, race, and gender (p < .05). Simple slopes of EIS will be estimated within each intersectional group (Black women, White women, Black men, White men) using the emtrends() function in the emmeans R package [85]. Planned contrasts will compare whether the EIS slope for Black women is significantly different from the slopes of the other three groups. Statistical significance will be evaluated at *p* < .05 (two-tailed).

## Supporting information

S1 File Supplementary materials for the EIS. (DOCX)
