# Supplementary material for: Capturing experienced invisibility among people with multiple stigmatized identities: The experienced invisibility scale protocol
Source: PLoS One. 2026 Oct 30;21(10):e0361028. doi: 10.1371/journal.pone.0361028 (PMC13722165; doi:10.1371/journal.pone.0361028)
Supplement: Supplementary file 1 [file pone.0361028.s001.docx]

**Supplemental Materials for**

*Capturing Experienced Invisibility Among People with Multiple Stigmatized Identities: The Experienced Invisibility Scale*

**Exploratory Factor Analysis (EFA) Using Sesko and Biernat (2025, Study 2) Data**

**Data Analysis**

***Initial Item Analysis***

Means, standard deviations, corrected item-total correlations, Cronbach’s alpha and McDonald’s omega if item deleted were examined for all items (see S1 Table). All items had similar means, SDs, similar ranges, and similar item-total correlations (.567-.819). All 13 items were subjected to an exploratory factor analysis, which guided item reduction.

**S1 Table. Original Experienced Invisibility Scale items in Sesko and Biernat (2025, Study 2), their range, means (sds), item total correlation, and total alpha if deleted.**

| **Item** | **N** | **Min** | **Max** | **Mean** | **SD** | **Item total correlation** | **Alpha if deleted** |
| --- | --- | --- | --- | --- | --- | --- | --- |
| EIS1 | 370 | 1 | 7 | 2.981 | 1.423 | 0.626 | 0.878 |
| EIS2 | 369 | 1 | 7 | 3.331 | 1.572 | 0.594 | 0.880 |
| EIS3 | 370 | 1 | 7 | 3.014 | 1.481 | 0.456 | 0.887 |
| EIS4 | 370 | 1 | 7 | 3.351 | 1.588 | 0.679 | 0.875 |
| EIS5 | 370 | 1 | 7 | 2.903 | 1.478 | 0.600 | 0.880 |
| EIS6 | 370 | 1 | 7 | 2.816 | 1.523 | 0.680 | 0.875 |
| EIS7 | 370 | 1 | 7 | 2.557 | 1.506 | 0.508 | 0.884 |
| EIS8 | 370 | 1 | 7 | 2.776 | 1.608 | 0.630 | 0.878 |
| EIS9 | 370 | 1 | 7 | 3.416 | 1.357 | 0.342 | 0.891 |
| EIS10 | 370 | 1 | 7 | 3.092 | 1.289 | 0.392 | 0.889 |
| EIS11 | 370 | 1 | 7 | 3.262 | 1.621 | 0.735 | 0.872 |
| EIS12 | 370 | 1 | 7 | 3.527 | 1.593 | 0.655 | 0.877 |
| EIS13 | 369 | 1 | 7 | 3.138 | 1.588 | 0.598 | 0.880 |

**Exploratory Factor Analysis**

Next, we conducted a parallel analysis using the psych package (Revelle, 2025) in R to determine how many factors to retain. Parallel analysis creates a set of random data with the same number of factors and number of participants as the original data set and generates a random factor structure. The factors from the actual data set are compared with those from the random data set. Factors with eigenvalues greater than the randomly generated factors are retained. The results indicated three factors with eigenvalues of 5.114, 0.599, and 0.432. However, given the relatively low eigenvalues of the second two factors, we also subjected the items to the Very Simple Structure (VSS) function which assesses how well a simplified factor solution (where each item loads on only 1 or 2 factors) can reproduce the correlation matrix of the data, thus giving a sense of which model gives a reasonable approximation of the true data. We used the VSS function to evaluate models ranging from one to three factors. The results were somewhat mixed; The VSS metric suggested a one factor model was simplest whereas the model fit characteristics suggested a three-factor model was most parsimonious (see S2 Table).

**S2 Table. Fit statistics based on Very Simple Structure (VSS) analysis.**

| **No. Factors** | **Chi² (*p*)** | **RMSEA** | **BIC** | **SRMR** | **VSS Fit** | **Residual Variance** |
| --- | --- | --- | --- | --- | --- | --- |
| 1 | 285 (*p* < .001) | 0.096 | -100 | 0.065 | 0.85 | 5.8 |
| 2 | 141 (*p* < .001) | 0.067 | -172 | 0.044 | 0.64 | 13.3 |
| 3 | 38 (*p* = .65) | 0.000 | -210 | 0.021 | 0.60 | 14.8 |

We therefore proceeded to run an exploratory factor analysis using maximum likelihood estimation, direct oblimin rotation, and specifying three factors. We used the guidelines by Costello and Osborne (2005) and Tabachnick and colleagues (2019) for avoiding multicollinearity to guide item reduction, which stipulates .32 as a cut off for item factor loadings. Upon examining the factor loadings, there was considerable conceptual overlap between items on Factor 2 (e.g., “Sometimes people who should know me don’t remember details about me.”) and 3 (e.g., “Sometimes people who should know me remember details about me that are untrue”) and one cross-loading item (i.e., “People often confuse me with others”) suggesting the three-factor solution is overly complex. We therefore proceeded to run an exploratory factor analysis using maximum likelihood estimation, direct oblimin rotation, and specifying two factors. The VSS results suggested that a two-factor model had superior fit to the one-factor model, making it a reasonable alternative (see S2 Table). Indeed, the 2-factor model provided a clearer solution with no cross-loadings (see S3 Table). Based on the results of the 2-factor EFA, we removed one item (EIS 9) that did not load on either factor (e.g., a factor loading <.32). The final scale consisted of 12 items (α = .89).

**S3 Table. Factor loadings for exploratory factor analysis in Sesko & Biernat (2025, Study 2)**

| **Item No.** | **Item** | **F1** | **F2** |
| --- | --- | --- | --- |
| 1 | My contributions to group discussions often go unnoticed. | 0.290 | **0.423** |
| 2 | Things I have said are sometimes misremembered as being said by someone else. | **0.662** | -0.020 |
| 3 | Sometimes people misremember me saying something, when it was actually said by someone else. | **0.562** | -0.060 |
| 4 | Sometimes people who should know me don’t remember details about me. | **0.752** | 0.022 |
| 5 | Sometimes people who should know me remember details about me that are untrue. | **0.690** | -0.032 |
| 6 | Sometimes people who should know me don’t recognize me. | **0.696** | 0.081 |
| 7 | People often confuse me with others. | **0.630** | -0.060 |
| 8 | I feel invisible. | 0.003 | **0.742** |
| 9 | I feel that people take what I have to say seriously. (reverse scored)* | 0.122 | 0.250 |
| 10 | I feel respected in most everyday interactions with others. (reverse scored) | -0.098 | **0.547** |
| 11 | In conversations with others, I often feel ignored. | 0.063 | **0.805** |
| 12 | I often feel as though people don’t listen to what I have to say. | -0.032 | **0.806** |
| 13 | Sometimes people forget that they’ve met me before. | **0.486** | 0.216 |
| *item not included in final scale | | | |

The first factor included seven items related to being misremembered (e.g., “Things I have said are sometimes misremembered as being said by someone else”) or forgotten (e.g., “Sometimes people forget that they’ve met me before”). We named this factor the “Unheard” subscale (α = .89), it accounted for 23% of the total variance.

The second factor contained five items related to feeling invisible (e.g., “I feel invisible”) or ignored (e.g., “In conversation with others, I often feel ignored). We named this factor the “Unnoticed” subscale (α = .83), it accounted for 19% of the total variance. Subscale scores were created by taking the mean of the items on each factor.

The EIS total score also demonstrated strong reliability (α = .89). Each subscale was significantly correlated with the EIS total score but the Unheard subscale was more strongly correlated with the EIS total score than the Unnoticed subscale, *t*(370) = -7.03, p < .001. See S4 Table for means, standard deviations, and the inter-correlation between the two subscales.

**S4 Table. Inter-correlations between Experienced Invisibility Scale total and subscale scores in Sesko & Biernat (2025, Study 2).**

|  | M | SD | Experienced Invisibility Total Score | Unheard Subscale | Unnoticed Subscale |
| --- | --- | --- | --- | --- | --- |
| Experienced Invisibility Total Score | 3.06 | 1.03 | -- | -- | -- |
| Unheard Subscale | 3.02 | 1.09 | 0.94 | -- | -- |
| Unnoticed Subscale | 3.13 | 1.16 | 0.89 | 0.67 | -- |

*all correlations significant at p < .001

**References**

Costello, A. B., & Osborne, J. (2005). Best practices in exploratory factor analysis: Four

recommendations for getting the most from your analysis. https://doi.org/10.7275/JYJ1-	4868

Hu, L., & Bentler, P. M. (1999). Cutoff criteria for fit indexes in covariance structure analysis:	Conventional criteria versus new alternatives. *Structural Equation Modeling: A	Multidisciplinary Journal, 6*(1), 1–55. https://doi.org/10.1080/10705519909540118

Revelle, W. (2025). *psych: Procedures for Psychological, Psychometric, and Personality	Research*. Northwestern University, Evanston, Illinois. R package version	2.5.6, [https://CRAN.R-project.org/package=psych](https://cran.r-project.org/package=psych).

Sesko, A. K., & Biernat, M. (2025). Experienced invisibility and well-being among people with	multiple-stigmatized identities. *Self and Identity*, *24*(7), 757–787.	https://doi.org/10.1080/15298868.2025.2526451

Tabachnick, B. G., Fidell, L. S., & Ullman, J. B. (2019). Using multivariate statistics (7th ed.).	Pearson.
